# The American and Colonial Journals: Pathology, Practical Medicine, and Therapeutics

**Published:** 1839-07

**Authors:** 


### II. THE AMERICAN AND COLONIAL JOURNALS.

### PATHOLOGY, PRACTICAL MEDICINE, AND THERAPEUTICS.

#### *On Narcotine as a substitute for Quinine in Intermittent Fever.* By Dr. O'SHAUGHNESSY.

ON the 4th of August, 1838, at the meeting of the Medical Society of Calcutta, Dr. O'Shaughnessy laid before the Society the details of thirty-two cases of remittent and intermittent fevers treated by narcotine as a substitute for quinine, and of which thirty-one were cured. The cases previously described in the first Report of the Pharmacopœia Committee were twenty-seven, making on the whole sixty, of which the narcotine was successful in all but two.

The cases now communicated were as follows:

Two cases by Dr. Goodeve. One of them, the case of the late deputy-collector of Chittagong. Quotidian of several months' standing, spleen enlarged. Quinine was used without success, although given in every possible form. Arsenic was then tried and checked the fever, but did much mischief to the patient's general health. Narcotine was then given, and with such success, that Dr. Goodeve concludes it thus: "I do not hesitate in saying that this patient owes his life to the remedy in question." The other case was a patient labouring under inflammation of the bowels at the same time, where the administration of quinine would have been inadmissible.

Three cases are reported by Dr. Smith, of Hidgelee, who adds, "As far as these three cases go, I cannot speak too favorably of narcotine, and am very desirous of trying it more extensively." Capt. Marshall, of Calcutta, communicated three cases of severe ague occurring among his servants; all were rapidly cured, and Capt. Marshall says, "It would be presumptuous in me to offer any opinion as to the virtues of narcotine; all I can say is, that if ever I am ill of fever I shall unhesitatingly and confidently prefer it to sulphate of quinine or any other medicine I know of."

Mr. R. O'Shaughnessy described the case of a man on whom he had operated for stone, and who was attacked by violent ague on the day of the operation. The ague returned next day at the same hour. Mr. O'Shaughnessy considered it unsafe to employ quinine under these circumstances, and had recourse to narcotine. Four doses of this medicine were given, and Mr. O'Shaughnessy states, "The fever did not return, the wound was not in the slightest degree affected; there was no excitement or headach produced. After he took the first dose he slept soundly, which he had not done the two previous nights, and he was discharged cured of the effects of the operation on the fourteenth day after its performance."

Mr. O'Brien, the apothecary of the Native Hospital, reported three cases, Mr. Evans one, the Pundit Modoosoodona Gupta one, all successfully treated. The pundit's patient laboured under dysentery at the same time.

Dr. J. Chapman, assistant-surgeon of the Calcutta General Hospital, related the case of a European who contracted violent remittent fever at Kedgeree on the 16th of July, and was received in hospital on the 19th. Quinine was used in the usual manner on the first remission on the 20th, and again on the 21st, but the symptoms were rather aggravated than improved. The narcotine was then given, and its use was speedily followed by a complete remission. From that time the fever did not return, with the exception of restlessness and slight headach on the evening of the 23d. On the 28th, all medicines were omitted, and the patient was discharged convalescent.

Dr. O'Shaughnessy further submitted two cases, treated in his own house among his servants, both of which were cured. Lastly, he communicated fifteen cases extracted from the journals of the Medical College Hospital. In five of these cases quinine and arsenic had failed, in eleven there was enlargement of the spleen or liver, in one inflammation of the knee-joint. Seven of these cases were remittents, and two of these had died. Of the two fatal cases one was admitted on the seventh day of violent fever and died next day. In the second (a child) the spleen, liver, pancreas, and mesenteric gland, were immensely enlarged, and the case hopeless from the beginning.

Dr. O'Shaughnessy added that, besides the sixty cases now recorded, more than 100 ague patients had been treated by his pupils and acquaintance with perfect success by this remedy.

[In a subsequent number of the India Journal the following letter appears, addressed to Dr. O'Shaughnessy, by Mr. Green, *Civil Surgeon, Howrah.*]

"I have now employed the narcotine in sixteen cases of remittent fever, and such is my opinion of the efficacy of the remedy, that in instances of fever, intermittents and remittents, in ordinary healthy subjects, and in whom there is no complication of severe organic disease, I give it with the full expectation of arresting the next periodic return of the fever. I have seen the result follow in ten of the cases of the fever alluded to. I consider narcotine a more powerful antiperiodic than quinine. The remedy does not act silently. I have observed a degree of general heat follow its use in the first instance, and, subsequently, perspiration, so that it appears to excite in the system a salutary and powerful counteraction as to stop the morbid concentration that issues in fever. I have not observed narcotine to lead to local organic disturbance in the cases in which I have used it. In short, even from my scanty experience, I consider the remedy an invaluable one."

*India Journal of Med. Science. Sept. and Nov.* 1838.

---

